# A home-visiting intervention targeting determinants of infant mental health: the study protocol for the CAPEDP randomized controlled trial in France

**DOI:** 10.1186/1471-2458-12-648

**Published:** 2012-08-13

**Authors:** Florence Tubach, Tim Greacen, Thomas Saïas, Romain Dugravier, Nicole Guedeney, Philippe Ravaud, Susana Tereno, Richard Tremblay, Bruno Falissard, Antoine Guedeney

**Affiliations:** 1 AP-HP, Hôpital Bichat, Département d’Epidémiologie et Recherche Clinique, 46 rue Henri Huchard, Secteur Claude Bernard, 75877 Paris Cedex 18, Paris, France; 2 Univ Paris Diderot, Sorbonne Paris Cité, Paris, France; 3 INSERM, CIE801, Paris, France; 4 Laboratoire de Recherche, Hôpital Maison Blanche, Paris, France; 5 Institut National de Prévention et d’Éducation de la Santé, Saint-Denis, France; 6 Université du Québec à Montréal, Montreal, Canada; 7 AP-HP, Hôpital Bichat, Service de psychopathologie du jeune enfant, de l’enfant et de l’adolescent, Paris, France; 8 AP-HP, Hôpital Hôtel Dieu, Centre d'Epidémiologie Clinique, Paris, France; 9 Univ Paris Descartes, Sorbonne Paris Cité, Paris, France; 10 INSERM U738, Paris, France; 11 Institut de Psychologie, Univ Paris Descartes, Sorbonne Paris Cité, Paris, France; 12 Laboratoire de Psychologie et Processus de Santé (LPPS-EA4057), Paris, France; 13 University College Dublin, Dublin, Ireland; 14 INSERM U669, PSIGIAM, Maison de Solenn, Paris, France; 15 AP-HP, Hôpital Bichat, Service de psychopathologie du jeune enfant, de l’enfant et de l’adolescent, Paris, France; 16 Univ Paris Diderot, Sorbonne Paris Cité, Paris, France; 17 INSERM U669, PSIGIAM, Maison de Solenn, Paris, France; 18 Institut Mutualiste Montsouris, Université René Descartes Paris 5, Unité Inserm 669, Paris, France

**Keywords:** Prevention, Mental health promotion, Home visiting, Infant mental health, Postnatal depression, Security of attachment and attachment disorganisation in infants, Randomized controlled trial

## Abstract

**Background:**

Several studies suggest that the number of risk factors rather than their nature is key to mental health disorders in childhood.

**Method and design:**

The objective of this multicentre randomized controlled parallel trial (PROBE methodology) is to assess the impact in a multi-risk French urban sample of a home-visiting program targeting child mental health and its major determinants. This paper describes the protocol of this study. In the study, pregnant women were eligible if they were: living in the intervention area; able to speak French, less than 26 years old; having their first child; less than 27 weeks of amenorrhea; and if at least one of the following criteria were true: less than twelve years of education, intending to bring up their child without the presence of the child’s father, and 3) low income. Participants were randomized into either the intervention or the control group. All had access to usual care in mother-child centres and community mental health services free of charge in every neighbourhood. Psychologists conducted all home visits, which were planned on a weekly basis from the 7^th^ month of pregnancy and progressively decreasing in frequency until the child’s second birthday. Principle outcome measures included child mental health at 24 months and two major mediating variables for infant mental health: postnatal maternal depression and the quality of the caring environment. A total of 440 families were recruited, of which a subsample of 120 families received specific attachment and caregiver behaviour assessment. Assessment was conducted by an independent assessment team during home visits and, for the attachment study, in a specifically created Attachment Assessment laboratory.

**Discussion:**

The CAPEDP study is the first large-scale randomised, controlled infant mental health promotion programme to take place in France. A major specificity of the program was that all home visits were conducted by specifically trained, supervised psychologists rather than nurses. Significant challenges included designing a mental health promotion programme targeting vulnerable families within one of the most generous but little assessed health and social care systems in the Western World.

**Trial registration:**

Current Clinical trial number is NCT00392847.

## Background

Infant mental health is a public health priority both internationally [1] and in France [2]. Mental health disorders in childhood have long term consequences throughout the lives of the individuals in question, their families and the social environment as a whole [3]. The prevalence of psychiatric disorders in infants is related to a variety of psychosocial vulnerability factors. More emotional and behavioural disorders are seen in children of young, first-time mothers [4,5]; in infants with low quality of home environment [6,7]; in children of mothers with postnatal depression [8,9] or who have less knowledge of infant development [10], less parenting skills [11] or insightfulness [12]; in children of mothers who smoke [13] or who have other health problems [14]; in situations of psychosocial parental stress [15] or less perceived social support [16]; in children of mothers showing attachment disorganization [17]; in preschoolers whose parents do not live together [18]; and in children of families of low socioeconomic status and educational level [18]. Furthermore, individual vulnerability appears to be linked to the accumulation of vulnerability factors rather than being a direct result of one particular factor [19].

Health promotion approaches have explored various strategies, including actions within educational settings as well as home-visiting programs, widely developed since the using a model developed by David Olds [14,20]. In the USA, services based on these latter programs are today supporting more than 500,000 families [21]. In Europe, they are increasingly being integrated into existing social and health care systems [22-24]. One of the main purposes of home-visiting programs is to act upon the determinants of child mental health, particularly by reducing the impact of social stressors on families [25-28], developing parenting knowledge and skills regarding child development [29] and promoting maternal health [30], for example in areas such as diet, sleep and substance misuse [29]. Typically, families receive home visits from qualified nurses or trained paraprofessionals, often from the communities being served, on a weekly or monthly basis, beginning during pregnancy and ending when the child is between two to five years old [31]. A number of studies have shown home visiting to be an effective strategy for improving child development and parenting in vulnerable families [25-28,32], and reducing the risk of child abuse [28]. However, in recent reviews of these programs, only one in two demonstrate significant and positive impacts on children [24,33], a phenomenon often attributed to variation in implementation practices [33,34], to difficulties engaging families [35] or to using home-visiting personnel who are insufficiently trained [30].

The CAPEDP (Compétences parentales et Attachement dans la Petite Enfance: Diminution des risques lies aux troubles de santé mentale et Promotion de la résilience - Parental Skills and Attachment in Early Childhood: reduction of risks linked to mental health problems and promotion of resilience) study is the first randomized, controlled trial assessing an evidence-based, home-visiting program in France. Towards the end of the 1990s, despite the existence in every neighborhood of government-run mother-child services as well as community mental health services for both children and adults, mental health professionals had been becoming increasingly concerned by the number of children living in vulnerable social situations being referred for care, typically for behavioural problems. An international conference [36] confronting evidence-based preventive programs from different national contexts provided the impetus for developing the first French home-visiting program specifically targeting infant mental health, in line with international best practice criteria [24,32,37,38], and adapted to the particularities of the French context. The resulting CAPEDP project involved designing, implementing and evaluating an early, long-term, supervised, home-based intervention targeting the determinants of infant mental health in families presenting multiple psychosocial vulnerability factors.

The CAPEDP program has two major specificities with regard to most other home-visiting programs. The first specificity was to address child mental health promotion in families that already have, at least theoretically, free access to one of the most extensive, comprehensive and longstanding social and health care systems in the Western World. Indeed, at the close of World War II, France developed nation-wide, community-based, mother-child support and prevention services with no out-of-pocket payment, known as the *Protection Maternelle et Infantile* (Mother and Child Protection Services or PMI). Today, mothers have direct access to PMI centres free of charge from the beginning of pregnancy right through to their child’s third birthday. France was also the first Western country to develop, across the country, free community mental health services for both adults and children. With regard to child and adolescent care, each community mental health service provides care with no out-of-pocket payment for a population area of an average 250,000 inhabitants and, although with limited resources, being able provide home visits if deemed necessary for the child’s mental health or safety. Families also automatically access specific social benefits (*allocations familiales*) provided by local government to help raise their children, if they accept to bring them in for a limited number of health check-ups and compulsory vaccinations. Furthermore, families identified by maternity ward staff as being particularly vulnerable will receive home visits by PMI nurses - although a 2002 study revealed that, in the majority of cases (60%), this happened only once and only 7% of these more vulnerable families received more than three home visits [39]. Although PMI nurses receive no specific training on mental health promotion or prevention and little organised psychological supervision, they can and do refer families directly to their local community child and adolescent mental health service. As for the PMI, the functioning, outcomes and cost/efficiency of these mental health services have undergone little systematic evaluation.

The second major specificity of the CAPEDP intervention was that the entire home-visiting program was conducted by qualified psychologists. It was hypothesised that professionals who were more highly trained in psychology would be more competent in recognizing the elements in play with regard to the determinants of infant mental health and more skilled in acting upon these determinants.

### Objectives

The aim of the CAPEDP trial was to evaluate, in young primiparous mothers presenting vulnerability factors associated with greater likelihood of child mental health disorders, the impact on infant mental health of a home-visiting program conducted by trained psychologists and targeting the major modifiable determinants of infant mental health.

The program evaluated three primary outcomes: child mental health at the age of two, as well as two potential mediating variables: maternal postnatal depression at three months postpartum and the quality of the home environment when the child was 12 months old.

Secondary objectives included evaluating the impact of the intervention on: maternal postnatal depression at 6 months postpartum, infant attachment quality at 18 months of age, the mother’s quality of attachment at her child’s second birthday, her knowledge and use of social, medical and educational support services, her perception of receiving support from her own personal network, her parenting perceptions and behaviour, the child’s psychomotor development, sustained withdrawal behaviour of the child at 18 months, the mother’s knowledge concerning child development, her parental stress concerning caring for her child, her access to training and employment, her own psychological health and, in the intervention group, the working alliance between the mother and the home-visiting team (Table 1).

**Table 1:** Outcome criteria and assessment schedule

**Instrument**	**Concept measured**	**Validation**	**Outcome assessor**	**Structure of instrument; scoring**	**Time of measurement**					
			**Place of evaluation**		**Prenatal**	**3 mths after birth**	**6 mths after birth**	**12 mths after birth**	**18 mths after birth**	**24 months after birth**
Edinburgh Post- partum Depression Scale (EPDS)	Pre and pospartum depression	Cox et al., 1987 [40]	Mother during home visits	10 items, 4 point-Likert scales (0–3)	X	X	X	X		
		French validation: Guedeney & Fermanian, 1995 [41]		Range 0–30						
				Higher scores indicate higher levels of depressive symptoms.						
Home Observation for the Measurement of the Environment (HOME) inventory	Quality of the home environment (quality and quantity of stimulation and support available to the child in the home environment)	Bradley & Caldwell, 1979 [42]	Assessment team during home visits	Designed for use during infancy (birth to age three).		X		X	X	
		French validation Gunning et al, 2004 [43]		45 items (yes/no response)						
				Six subscales:						
				Parental Responsitivity (11 items)						
				Acceptance of the child (8 items)						
				Organization of the environment (6 items)						
				Learning Materials (9 items)						
				Parental Involvement (6 items)						
				Variety in Experience (5 items)						
				Highest scores for each subscale indicate greater environment.						
Child Behavior Checklist 1½-5 (CBCL 1½-5)	Child behavioural disorders	Achenbach 2009 [44]	Assessment team during home visits	100 items, Likert scales (0–2)						X
				Range 0–200						
		French validation: Ivanova et al, 2010 [45]								
				A total score and 7 syndrome scales:						
				Emotionally Reactive (0–18, clinical range (CR) >8)						
				Anxious/Depressed (0–16, CR>8)						
				Somatic Complaints (0–22, CR>6)						
				Withdrawn (0–16, CR >5)						
				Sleep Problems (0–14, CR>8)						
				Attention Problems (0–10, CR>6)						
				Aggressive Behaviour (0–38, CR>24)						
Attachment Q –Sort (AQS)	Child’s attachment	Waters & Deane, 1985 [46]	Assessment team during home visits	Two raters assess each situation, during a home visit of two hours.					X	
		French translation made and validated by a panel of infant mental health experts								
				90 items						
				Correlation with typical secure pattern:						
				Insecure attachment if < 0.35						
				Secure attachment if ≥0.35						
Vulnerable Attachment Style Questionnaire (VASQ)	Mother’s attachment	Bifulco et al., 2003 [47]	Mother during home visit	22 items, 5-point Likert scale (1–5).	X					X
				Range 0–110						
				1 global scale: Vulnerability (0–110, vulnerability if ≥ 57)						
				2 sub-scales:						
				Insecurity (range 0–60, insecure if > 30)						
				Proximity seeking (range 0–50, proximity seeking if ≥ 27)						
Services Questionnaire	Use of social and / or medical services	Specifically designed for this research	Assessment team during home visits	Description of the use of 27 social and / or medical services	X	X	X	X		X
Social Support Interview (SS-A; SS-B)	Perception of social support received and level of satisfaction from the social network	Designed for this research based on Vaux, 1988 [48]	Assessment team during home visits	6 items	X	X		X		X
				Description of social support structure (emotional, material, financial, socialization, valorization)						
Parental Cognitions and Conduct Toward the Infant Scale (Pacotis)	Parenting: mother perception of her attitude and behaviour towards her child, of her competence or incompetence and of her emotional investment of the child.	Boivin et al., 2005 [49]	Mother during home visit	17 items, numeral rating scales (0–10)		X		X		
				3 sub-scales (mean score of items)						
				Parental self-efficacy; Perception of parental impact; Hostile-reactive parenting behaviour						
				Higher sub-scale scores indicate lower parental self-efficacy, perception of less parental impact on the child's behaviour, and higher parental use of hostile-reaction behaviour.						
Brunet-Lézine developmental test (BL-Revised)	Child development (0 to 5 years)	Brunet & Lezine, 1965 [50]	Assessment team during home visits	Developmental age (developmental quotient)			X	X		
				4 dimensions:						
				Language						
				Motor gross						
				Motor fine						
				Social relationships						
				30 item test scored partly						
				on observation, partly on questions to parents						
		Original scale in French								
Alarm Baby Distress scale (ADBB)	Sustained withdrawal behaviour	Guedeney & Fermanian, 2001 [51] in France	Assessment team during home visits	8 items, 5-point Likert scale (0–4), Range 0–32					X	
				Withdrawal behaviour if ≥ 5						
Knowledge of Infant Development Inventory (KIDI)	Mother’s knowledge of infant development	McPhee, 1981 [52]	Mother during home visit	48 items (−1, 0, 1)	X	X				X
				Range −48 to 48						
				Highest scores indicate better knowledge.						
Parental Stress Inventory (PSI)	Parental stress	Abidin & Wilfong, 1989 [53]	Mother during home visit	24 items, Likert scales (1–5)		X	X	X		X
				Two subscales (mean score of items):						
				Parental stress						
				Dysfunctional interaction						
				Higher scores indicate greater parental stress.						
Symptom Check-list (SCL-90)	Mother’s psychological disorders	Derogatis, 1994 [54]	Mother during home visits	90 items, 5 point-Likert scales (0–4)	X	X				X
				10 subscales:						
				Somatization (0–48)						
				Obsessive-Compulsive (0–40)						
				Interpersonal Sensitivity (0–36)						
				Depression (0–52)						
				Anxiety (0–40)						
				Hostility (0–24)						
				Phobic Anxiety (0–32)						
				Paranoid Ideation (0–24)						
				Psychoticism (0–40)						
				Other symptoms (0–28)						
				Higher scores indicate greater clinical impairment.						
Working Alliance Inventory (WAI)*	Working alliance between the mother and the CAPEDP intervention psychologist	Horvath & Greenberg, 1989 [55]	Mother during home visits	12 items, Likert scale (1–7)		X	X	X	X	X
				Range 12–84						
		French validation Guedeney et al, 2005 [56]								
				Higher scores indicate better working alliance						
Strange Situation Procedure (SSP)^#^	Infants’ Attachment	Ainsworth & al, 1978 [57]	Assessment team viewing video of infant in attachment laboratory	Categorical				X		
								(12 to 15 mths)		
				Attachment:						
				Secure						
				Insecure-Avoidant						
				Insecure-Ambivalent/Resistant						
				Disorganised/Disoriented						
Atypical Maternal Behaviour Instrument for Assessment and Classification (AMBIANCE) ^#^	Maternal Disruptive Behaviour	Lyons-Ruth, Bronfman, & Parsons, 1999 [58]	Assessment team viewing video of mother in attachment laboratory	Categorical:				X		
				Maternal Affective Communication not Disrupted if < 5				(12 to 15 mths)		
				Maternal Affective Communication Disrupted if ≥ 5						
				2 Sub-styles:						
				Withdrawal /Disoriented						
				Hostile/Role Confusion						
Insightfulness Assessment (IA) ^#^	Maternal Auto-reflexive function	Oppenheim & Koren-Karie 2002 [59]	Assessment team viewing video and interview of mother in Attachment laboratory	10 scales, giving way to a 4 category classification:				X		
								(12 to 15 mths)		
				Positive Insight						
				One-sided						
				Disengaged						
				Mixed						

* in the intervention group only.

^#^ in the CAPEDP-A subsample only.

### An ancillary study: the CAPEDP-A Study

Assessment of attachment security and caregiver behaviour being particularly complex from a procedural point of view, an ancillary study involving a subsample of the CAPEDP population was designed to investigate this particular point: the CAPEDP Attachment (CAPEDP-A) Study. The objectives of this ancillary study were to assess the impact of the CAPEDP intervention in terms of increasing infant attachment security and maternal reflexive ability and reducing infant attachment disorganisation and maternal disorganizing behaviour when the child was from 12 to 15 months old.

## Methods/design

The CAPEDP Study is a prospective, randomized controlled, multicenter trial with two parallel arms comparing the CAPEDP intervention to usual care. The trial used Prospective Randomized Open Blinded Endpoint (PROBE) methodology with a 27-month follow-up. Usual care involved access to the PMI and community mental health networks with no out-of-pocket payment, free antenatal maternity screenings, and a variety of social benefits, as described above. The intervention group benefited additionally from the CAPEDP home-visiting program (see below).

### Study population

Eligibility criteria limited participation to mothers in situations of medium to high vulnerability with regard to their future child’s mental health. All consecutive women consulting in the second trimester of pregnancy (from 12 to 27 weeks of amenorrhea) in ten public maternity wards were assessed for eligibility. Pregnant women were eligible if they were: living in the intervention area (Paris and its inner suburbs); sufficiently fluent in French to give valid informed consent, benefit from the intervention and participate in assessment sessions; less than 26 years old; first time mothers; less than 27 weeks pregnant at their first home visit assessment session; eligible for legal national health insurance or its equivalent for non-French participants (as required by French law on clinical research). They also had to declare at least one of the three following criteria: 1) having less than twelve years of education, 2) intending to bring up their child without the presence of the child’s father, and 3) having low income, defined as being eligible for French social welfare health insurance (*Couverture Maladie Universelle Complémentaire*) i.e. with an income less than or equal to 850 euros a month or, for undocumented migrants, Government Medical Aid (*Aide médicale d’Etat*).

Exclusion criteria were: women who would be impossible to follow up, such as Roma, gypsies, travelers, the homeless, or temporary refugees; women already receiving sustained social or medical care for other reasons than the above inclusion criteria (such as addictions or mental or physical disorders requiring close long-term follow-up); and women who did not consent to participate.

Participation in the study was proposed to eligible women in the waiting rooms of each maternity hospital, prior to a prenatal appointment. During this interview or at a second appointment if she asked for more time to make up her mind, the future participant signed the informed consent form.

#### CAPEDP-A subsample

When their child reached 12 months of age, all families participating in the main CAPEDP trial were consecutively invited to participate in the CAPEDP-A study. After receiving information about the study, and if they accepted to participate with their child, mothers signed an informed consent form and an appointment was given to them for a two hour assessment procedure within the following fortnight. Inclusion was terminated when the required 120 mothers had accepted to participate with their child. Mothers received 50 euros gratification for participating in the CAPEDP-A assessment.

### Randomisation and masking

After completing baseline screening and informed consent procedures, participants were randomly assigned in a 1:1 ratio to either the CAPEDP intervention or the usual care group using a computer-generated randomisation sequence, stratified by recruitment centre, with random block sizes of 2, 4 or 6 participants. This sequence was centrally generated by the Clinical Research Unit of Bichat Hospital, Paris, France. Assignment of participants was concealed using centralized randomisation through fax in the Clinical Reseach Unit. Investigators thus had no knowledge of the next assignment in the sequence in this open label trial. Investigators, psychologists performing the CAPEDP intervention and participants were blinded to assignment before, but not after, randomisation, as per the open-label design. However, in accordance with PROBE methodology, the outcome assessors were blinded to assignment and no investigators, psychologists or participants had any knowledge of aggregate outcomes at any point during the course of the study.

### The CAPEDP intervention

The intervention sought, where possible, to act upon the major modifiable determinants of infant mental health from the third trimester of pregnancy to the child’s second birthday. Intervention strategies were based upon three main theoretical concepts: parental empowerment, attachment security and Fraiberg’s developmental guidance and *Ghost in the Nursery* concepts [60]. With regard to parental empowerment, the programme manual used Rappaport’s [61] definition of empowerment as being made up of four components: participation, competence, self-esteem and personal and collective consciousness. The intervention thus specifically targeted mothers’ use of their personal community networks, their parenting skills, and their knowledge and use of available resources within the generous French social and health care context. Bowlby [62] defines attachment as a primary drive, a search for security through physical closeness when the child is in distress due to pain, hunger, stress, fear or separation. Depending on their mothers’ sensitivity and responsiveness to signals of distress, children develop, as early as 12 months of age, different styles of attachment. Secure attachment has been linked with increased resiliency, whereas insecure attachment and, even more so disorganized attachment, are associated with increased internalized and externalized psychopathology [63,64]. Increasing security of attachment and decreasing attachment disorganization were key intervention targets. Finally, providing social and emotional support within a solid working alliance to isolated, young mothers, often with difficult childhood experiences and high levels of postnatal depression, helped to connect the mothers’ past experiences with their behaviour when interacting with their child, thus uncovering potential ‘*Ghosts in the nursery’* and helping young mothers explore new ways of relating to their children.

As indicated above, a major specificity of the CAPEDP intervention was that the home-visiting intervention and its evaluation were entirely conducted by trained psychologists. Eleven psychologists were recruited and assigned to either the intervention team (n = 7) or the assessment team (n = 4). All home-visiting psychologists received intensive training on implementing the CAPEDP intervention. Using the theoretical bases described above, the intervention was tailored to target, in terms of maternal empowerment, mothers’ knowledge and skills with regard to parenting, their ability to make the most of the care system, and their involvement with their own personal and local community networks. In terms of mother-child relationships, increasing security of attachment and decreasing attachment disorganization were key intervention targets. Finally, home-visiting psychologists received training on providing social and emotional support to the mothers within a solidly constructed working alliance, and helping mothers connect their past experiences with their current behaviour when interacting with the child, identifying *ghosts in the nursery* and exploring new ways of relating to their children. Manualised, but tailored to each family’s needs, the intervention targeted objectives specific to each child development period: prenatal, 0 to 3 months, 3 to 6, 6–12, and 12 to 24 months. The manual drew from Weatherston’s work on home-visiting and reflective supervision [65], the Florida State *Partners for a Healthy Baby Home Visiting Curriculum *[66], and the *Steps Towards Effective Enjoyable Parenting* (STEEP) attachment-based program [67]. Extensive use was made of McDonough’s developmental guidance approach through the use of video clips, filmed and discussed with the mothers [68]. Visits included showing mothers films on different aspects of parenting, from delivery through the different stages of child development. Further details of the content of the intervention can be found elsewhere [69]. Training also included a specific section on ethics and research procedures.

The program was designed for psychologists to visit families six times during the antenatal period, eight times in the first three months of the child's life, 15 times when the child was between 4 and 12 months of age and another 15 times during the child's second year, resulting in a total of 44 home visits during the whole intervention. Between visits, phone calls could be made as often as necessary.

Each psychologist doing home visits had weekly individual supervision with a member of a team of psychiatrists and psychotherapists, as well as group supervision with the main investigator (AG) to assess and react to situations of danger whether it be for the children (abuse, neglect, developmental delay, etc.) or their mothers (psychopathology requiring specific help, suicidal thoughts, special health needs etc.). Home visitors were encouraged to refer to the main investigator if they felt the slightest danger, if they felt distressed or if they felt that a situation was getting out of hand in any way.

### Description of the control group: usual care

Usual care, as described above, involved free access to the PMI network and to community mental health services, free antenatal maternity screenings by local GPs, extensive social security allowances and facilitated access to housing.

### Procedures and outcome measures

Families in the control group received usual care and seven assessment home visits across the trial period. The intervention group, in addition to usual care and assessment visits, received the CAPEDP home-visiting program. Assessment visits were conducted during specific home visits by a team of four trained and supervised psychologists, working independently from the psychologists performing the CAPEDP intervention, and with no prior knowledge of whether the families they were assessing were in the intervention group or the control group. For each family, seven home-based assessment visits were scheduled across the trial period, at the 27^th^ week of pregnancy, and then when the child was 3, 6, 12, 18 and 24 months old. All measured outcome criteria and the month at which these were measured are presented in Table 1. The assessment team received specific training on the use of all assessment instruments. Individual and group supervision was provided for all members of the assessment team, to give them support when faced with difficult situations during evaluation. Evaluators who observed significant problems or risk situations, either for the mother or for the child, in any family were instructed to seek immediate advice from the principal investigator. Families were referred to immediate care and support if necessary.

At baseline and, if appropriate, at follow up visits, the following data were collected: socio-demographic data including standard questions on age, sex, marital status, ethnicity, household composition, composition of the mother’s family, characteristics of the partner and, if different, the child’s father, whether the pregnancy was desired or not, number of years of education, educational level achieved, employment status, and income; health variables including mothers’ perceived state of health, and tobacco, alcohol or drug consumption. Neonatal data concerning the child and childbirth were collected while the mother was still in the maternity ward after giving birth.

As mentioned above, the study had three primary objectives: child mental health at the age of two as well as two potential mediating variables: maternal postnatal depression at three months postpartum and the quality of the home environment when the child was twelve months old.

Child mental health at the age of two was assessed using the Child Behavior Checklist 1½-5 (CBCL 1½-5) [70]. This instrument is widely used to assess psychopathology in infants and toddlers. A recent validation study in 23 societies, including the French translation and validation used in the present study, confirmed transcultural validity [45]. It is a 100-item scale, divided into seven syndrome subscales.

Maternal postnatal depression was assessed using the Edinburgh Postnatal Depression Scale (EPDS) [40]. This is a 10-item self-report questionnaire designed to be completed in the presence of an observer. It is valid for assessing both pre- and postpartum depression. The EPDS has been validated in a French population [71]. Different EPDS threshold scores were used to distinguish between depression and major depression.

The quality of the home environment when the child was 12 months old was assessed using the Home Observation for the Measurement of the Environment (HOME) [42]. This is a well-known and widely-used scale assessing the quality and quantity of stimulation and support available to a child in the home environment. As recommended, scoring was conducted during a home visit which did not have scoring the HOME as its unique objective.

Secondary outcome measures are described in Table 1.

The CAPEDP-A study assessed infant attachment quality, maternal disrupting behaviour and parental reflexive capacity using the Insightfulness Assessment (IA) interview.

Infant attachment quality was assessed using both the Strange Situation Procedure (SSP) [57] in our laboratory and the Attachment Q- Sort procedure [46], during a home visit. The SSP was used only in the CAPEDP-A subsample because of its complexity and time-consuming scoring. The procedure took place in an attachment assessment laboratory created specifically for this purpose in the research centre. The SSP proposes a fixed sequence of eight episodes, each lasting three minutes, designed to activate and/or to intensify the attachment behaviour of one year old infants. The procedure involves two brief separations and two reunions between the infant and their attachment figure, in the present case, their mother. Each procedure was video-taped. The procedure was coordinated by a senior psychiatrist trained in the use of the instrument and its coding. Psychology residents acted the roles of the strangers. The assessment of infant attachment quality using the SSP identifies three categories of attachment patterns. The insecure-avoidant group (A) is characterized by the infant avoiding manifesting attachment behaviour towards the attachment figure. The secure group (B) includes infants who evidence active proximity-seeking and interaction with the attachment figure, especially in reunion episodes. The insecure resistant/ambivalent group (C) is characterized by the coexistence of active contact resistance behaviour and proximity-seeking or contact-maintaining behaviour with the attachment figure. Coding of the procedure was made on video recordings by independent raters who were blinded to randomisation groups to reduce subjectivity-related variability. One assessment team and three coding teams were established. Coders had no direct contact with any dyad and were not aware of the group (intervention or control) to which the dyads belonged. Each measure was coded by separate coders. Furthermore, two independent coders coded a random selection of 30% of the cases. Inter-observer concordance calculated using Cohen’s Kappa coefficient was satisfactory (kappa = 0.79). Disagreements were discussed and a consensus category was attributed to the dyads in question. The SSP recordings were also used to assess disorganized attachment. Assessment was conducted by one of the authors (ST), who was specifically trained and validated for this coding.

Maternal disrupting behaviour was assessed using the Atypical Maternal Behavior Instrument for Assessment and Classification (AMBIANCE) scale [58]. This 5-dimension scale, scored on the SSP procedure video, measures a broad range of maternal behaviors that can be potentially disorganizing to infants' attachment. Higher scores reflect higher levels of maternal disorganising behaviour when attachment issues are raised. The scoring team was trained by Karlen Lyons Ruth and Elisa Bronfmann, the creators of the AMBIANCE scale, in a training session in Paris in 2008 and demonstrated full reliability. AMBIANCE coders were different from SSP coders and blind to the randomisation group of the families being assessed.

Parental reflexive capacity was assessed using the Insightfulness Assessment (IA) interview [59]. This is a semi-structured interview concerning the mother’s capacity to see things from the child’s point of view. It is assessed after she has viewed a video clip of herself with her child (nappy changes, free play, feeding or SSP). The IA is scored using ten sub-scales, and results in a classification into four categories. Each dyad was video-taped for SSP, nappy change, feeding and free play. The four IA raters achieved reliability on IA rating during a specific 2007 training with the team that created the instrument. Raters for IA were blind to the randomisation group of the dyad. The IA interviews took place at the same time as the SSP, when the infant was from 12 to 15 months of age.

Psychologists were asked to keep case notes on each home visit. These will be used to evaluate the frequency of the different themes that were notified as discussed during the home visit, the home visitor’s subjective perception of the visit, and the extent to which the home visitors’ preoccupations during that visit corresponded to the intended intervention.

### Adherence and withdrawal

Participants were informed that they could withdraw at any time for any reason. In order to optimise adherence to the intervention, families were reminded of upcoming visits by phone or with a text message. Missed home visits were rescheduled within the following week. Home visitors were also encouraged to maintain telephone contact with families between visits. Families that regularly missed home visits or did not respond to phone calls continued to receive regular calls at least once a fortnight from their home visitor. These calls continued through to the end of their planned participation in the study. Letters were regularly sent to each family that had not been in direct contact with their home visitor for a period of over three months without giving news. All participants, including those lost to follow up by the intervention team, were contacted by the evaluation team at every assessment point (at 3, 6, 12, 18, 24 months). In cases where families accepted phone contact but were reticent to receiving any further home visits, assessment took place over the telephone, except for those instruments that required direct observation. If phone contact proved to be impossible, questionnaires were sent to families via the post.

### Statistical analysis

#### Determination of sample size

The trial was designed to establish whether the CAPEDP intervention was superior to usual care in terms of postnatal maternal depression prevention as assessed using the EPDS, the quality of the home environment assessed with the HOME, and child psychopathology, assessed using the CBCL 1½-5.

Regarding the prevention of postnatal maternal depression, assuming a mean of 12.1 on the EPDS (SD 4.6) for the usual care group [41], 113 participants per study group would be sufficient to detect a 2-point decrease in the EPDS with 90% power at a 2*-*sided significance level of α = 5%.

Regarding quality of the home environment, assuming a mean of 25.5 on the HOME (SD 4.3) for the usual care group [72], 99 participants in each study group would provide 90% power at a two-sided α = 5% to detect a 2-point increase in the HOME.

Regarding infant psychopathology, assuming a mean score of 48.5 on the CBCL 1½-5 (SD 8.95) for the usual care group [73], 189 participants in each study group would provide 90% power at a two-sided α = 5% to detect a 3-point difference in the CBCL 1½-5.

To account for possible patients lost to follow up and have sufficient power to answer all three primary objectives, the project planned to recruit 440 families.

#### Statistical analysis

The data will be summarized using mean, median, standard deviation and range for continuous data and counts or percentages for categorical data.

##### Primary analyses

The data will be analyzed according to the modified intention-to-treat principle: all participants are taken into account within their particular assignment group whatever might have happened during the study, and all randomized participants that have at least one assessment visit within the first year of follow-up will be included for analysis. Missing data will be handled using multiple imputation and sensitivity analyses will be conducted. The between-group absolute differences in the EPDS score when the child is 3 months old, the HOME score at 12 months and the CBCL score at 24 months will be analyzed using Student’s t tests.

##### Secondary analyses

Data that are normally distributed will be analyzed using Student’s t test for continuous data, and chi-square test for categorical data, if the corresponding assumptions are fulfilled. If not, appropriate non-parametric methods will be used. In the intervention group, compliance to the CAPEDP program will be described by the proportion of planned home visits that actually took place performed reported to those scheduled. All statistical analyses will be considered significant at the 5% confidence limit using 2-sided tests. SAS software (version 9.1) will be used for statistical analyses.

More than 5,000 home visits were scheduled. A qualitative and quantitative analysis is being performed on the home visitors’ case notes to evaluate the extent to which the intended intervention program was effectively implemented or not. Case notes are analysed with regard to the frequency of themes that were declared as discussed during each home visit as well as the home visitors’ subjective perceptions of the visit.

### Ethical principles and safety

The study was designed and carried out in accordance with the principles of the Declaration of Helsinki, 5^th^ revision [74]. The study protocol was approved for all centres by the Institutional Review Board ‘Comité de Protection des Personnes Ile de France IV’ (IRB authorisation 2006/37). Written informed consent was obtained from all participants before inclusion. The trial is registered as ClinicalTrials.gov number *NCT00392847.*

## Discussion

The CAPEDP study is the first large-scale attempt in France to assess the impact of a home-visiting program on infant mental health in a highly vulnerable urban sample, thus replicating to a certain extent Old’s Elmira study in the US, but with a more systematic focus on the major modifiable determinants of infant mental health, particularly with regard to attachment security, the development of healthy mother-child relationships, pre and postnatal maternal depression, parenting skills and the quality of the home environment, and knowledge of community health and social care resources. A major specificity of the program is that the French context is characterized by easy access for all to community-based perinatal medical services, mental health services and social support, even for illegal immigrants. Hence, a significant aspect of the intervention was to encourage and help mothers to use the existing care system.

The second major specificity of the CAPEDP intervention was that, unlike other prevention programs based on home-visiting by nurses or trained community members, the entire home-visiting intervention was conducted by qualified psychologists, with the hypothesis that professionals who are more highly trained in psychology will be more skilled in identifying and acting upon the different potential determinants of infant mental health in multi-risk situations.

A limitation of the present study is that the control group cannot be considered to have received no intervention, on the one hand because “care as usual” is particularly generous in the French health and social care system and, on the other, due to the fact that the evaluation process could well be considered to be an intervention in itself, creating a Hawthorne effect. Furthermore, attrition is well-known to be a major challenge for home-visiting programs in vulnerable families [75-77]. In spite of considerable efforts to optimise adherence, CAPEDP is proving to be no exception to the rule. This is all the more so in that, in the French context, access to such a generous care system may also have an unfavourable impact on adherence, with mothers considering that they have all the help they need or preferring local community support to potentially stigmatising home visits by psychologists.

Another limitation is that randomisation was performed at inclusion, rather than after the first assessment visit. A significant number of randomized women withdrew their consent to participate before any data had been collected. This will necessarily result in a modified intent to treat analysis, a classical intent to treat analysis being impossible.

Furthermore, it must be underlined that women who were not fluent enough in French to give informed consent to participate or who were already receiving other types of clinical interventions were not eligible for inclusion in the present study. Similarly families for whom home-visiting would be impossible, for example Roma, gypsy or traveller families, transient refugees or homeless women were excluded. Generalising from results from this study to all mother-child dyads in multi-risk social situations will therefore be hazardous.

## Competing interests

The authors have declared no competing interests.

## Authors’ contributions

FT conceived and designed the study, participated in the coordination of the study, contributed the analysis plan and supervised statistical analysis, generated the randomisation, and drafted the manuscript. TG conceived and designed the study, participated in the coordination of the study, contributed the analysis plan and drafted the manuscript. TS conceived and designed the study, participated in the coordination of the study and contributed the analysis plan. RG conceived and designed the study, participated in the coordination of the study and contributed the analysis plan. PR participated in the design of the study. ST participated in the design and coordination of the CAPEDP –A ancillary study and contributed its analysis plan. RT partipated in the conception of the study. BF gave methodological and editorial support. AG conceived and designed the study and participated in the coordination of the study and drafted the manuscript. NG contributed to study design, participated in the study conception and read and approved the final version of the article. All authors read and approved the final manuscript after revising it critically for important intellectual content.

### Funding and sponsoring

The study received ethics approval (CPP Paris Saint Louis, advice n° 2006/37) and grants from major funding bodies: CAPEDP was supported by research grants from the French Ministry of Health Programme Hospitalier de Recherche Clinique (PHRC: AOM05056), the Institut National de Prévention et d’Education à la santé (INPES: DAS 08/2006 DAS 018/09 DAS 084/10), the Institut de Recherche en Santé Publique (IReSP: REV0702). The clinical trial number is NCT00392847.

The sponsor was the Département à la Recherche Clinique et au Développement, Assistance Publique–Hôpitaux de Paris.

## Pre-publication history

The pre-publication history for this paper can be accessed here:

http://www.biomedcentral.com/1471-2458/12/648/prepub
